# Branch retina vein occlusion combined with angle-closure glaucoma is associated with a mutation in BEST1: a case report

**DOI:** 10.1186/s12886-022-02504-w

**Published:** 2022-06-29

**Authors:** Xue Yin, Qinhua Cai

**Affiliations:** Department of Ophthalmology, Affiliated First Hospital of Soozhow University, Shizi Street 188, Suzhou, 21006 Jiangsu Province China

**Keywords:** Autosomal recessive bestrophinopathy, BEST1, Angle-closure glaucoma, Branch retina vein occlusion, Case report

## Abstract

**Background:**

It is rare for a patient to be diagnosed with branch retina vein occlusion (BRVO), angle-closure glaucoma (ACG) and autosomal recessive bestrophinopathy (ARB). ARB is strongly associated with ACG. Although glaucoma is a significant risk factor for RVO, there is a plausible relationship between ACG and BRVO. To discuss correlation of these diseases is necessary.

**Case presentation:**

The genetic testing and medical treatment of a patient with ocular fundus diseases and ACG were recorded. We present a 47-year-old male patient with BRVO who was diagnosed with angle-closure glaucoma and a homozygous mutation of c.140G > A (p.R47H) in BEST1. Intravitreal ranibizumab was administered in combination with three antiglaucomatous eyedrops to lower intraocular pressure (IOP) in the right eye. One month later, BCVA improved to 0.3. IOP was controlled at 13 mmHg.

**Conclusions:**

ACG was likely combined to ARB, while there’s a plausible relationship between ACG and BRVO.

## Background

Mutations in the *BEST1* gene are associated with a wide range of ocular phenotypes, including autosomal recessive bestrophinopathy (ARB). ARB is clinically characterized by central visual loss from subretinal fluid or macular edema and characteristic retinopathy consisting of punctate flecks [1]. Recently, ARB has been associated with hypermetropia and angle-closure, which are predispositions to narrow-angle glaucoma [2].

There is no denying that glaucoma coexists with retinal hemodynamic abnormalities. Additionally, it has been suggested that the pathogenesis of retina vein occlusion (RVO) is associated with glaucomatous anatomic changes [3]. Here, we present a case of angle-closure glaucoma (ACG) associated with a BEST1 homozygous mutation of ARB and a combined branch retina vein occlusion (BRVO) and analyze the correlation of these diseases.

## Case presentation

**Fig. 1:**
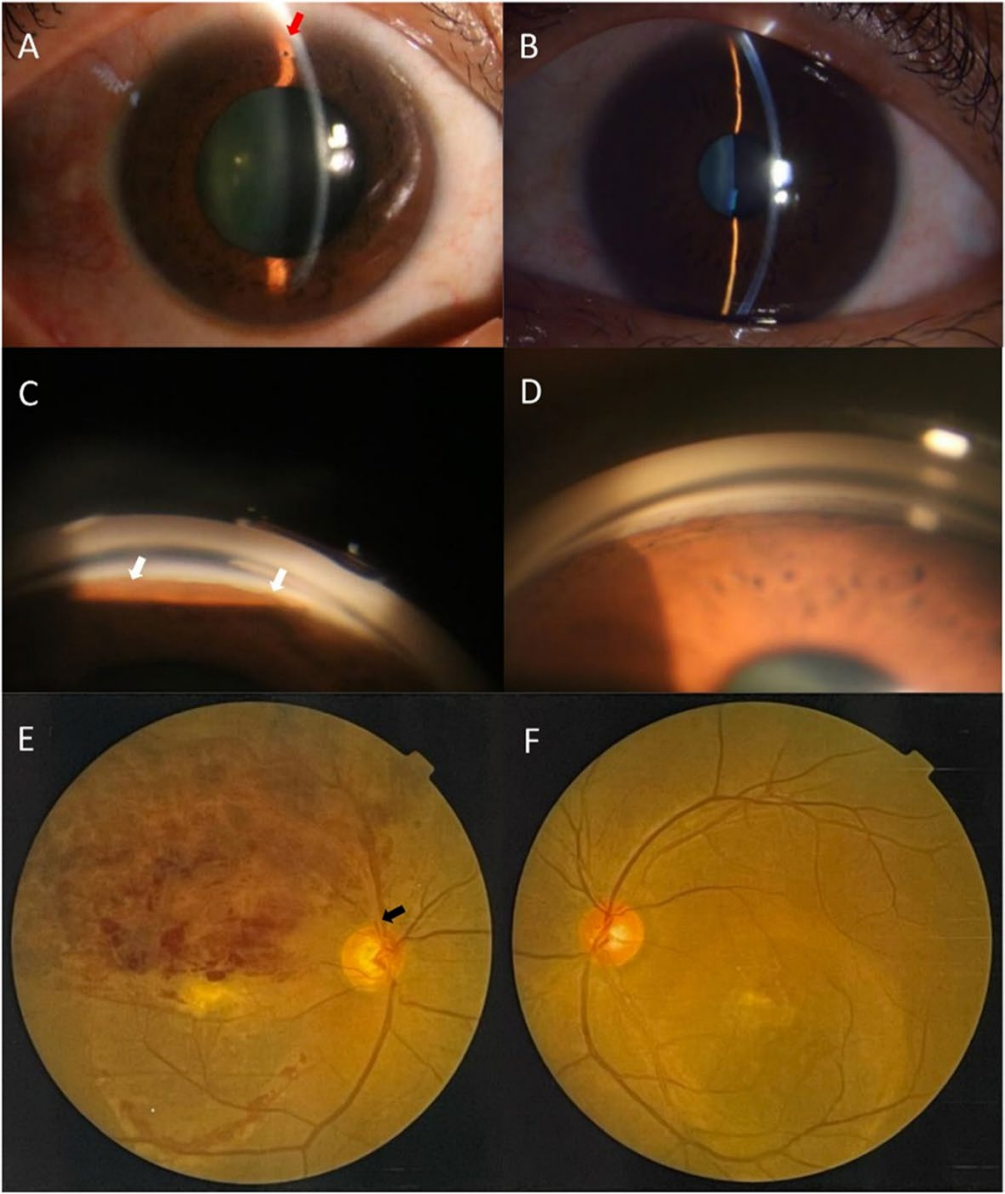
Photos of the patient. **A** Ocular anterior-segment image of the patient showing a peripheral iris bombe in the right eye (red arrow). **B** Ocular anterior-segment image of left eye. **C** Slit-lamp anterior-segment photography of the peripheral chamber. Anterior synechia in the right eye (white arrow). **D** Photography of the peripheral chamber of left eye. **E**, **F** Color fundus photographs of the right eye and left eye showing vitelliform deposits in macular and retinal hemorrhages. The superior temporal vein is occluded in the optic cup (black arrow)

A 47-year-old male presented to the First Affiliated Hospital of Suzhou University with a 2-week history of worsening visual acuity in the right eye. The patient stated that he had not any systemic diseases including hypertension and diabetes. The best corrected visual acuity was 0.08 in the right eye (OD) and 0.3 in the left eye (OS). In the right eye, the peripheral anterior chamber depth (ACD) was shallow. The pupil was dilated and was not sensitive to light (Fig. 1A). Shallow peripherally anterior chamber was observed in left eye (Fig. 1B). A circumferential grade IV in Scheie grading was observed in static gonioscopy, and almost 5 clock hours open in the right eye was observed in dynamic (Fig. 1C). No significant peripheral angle chamber synechia was found in the left eye (Fig. 1D). Fundus examination revealed superior intraretinal hemorrhages with an enlarged optic disc. Intraocular pressure (IOP) were 33 mmHg OD and 17 mmHg OS. The ACD was 1.79 mm in the right eye and 2.27 mm in the left eye. The axial length (AL) was 22.02 mm in the right eye and 22.65 mm in the left eye. At the first visit, the patient was diagnosed with BRVO and ACG.

**Fig. 2:**
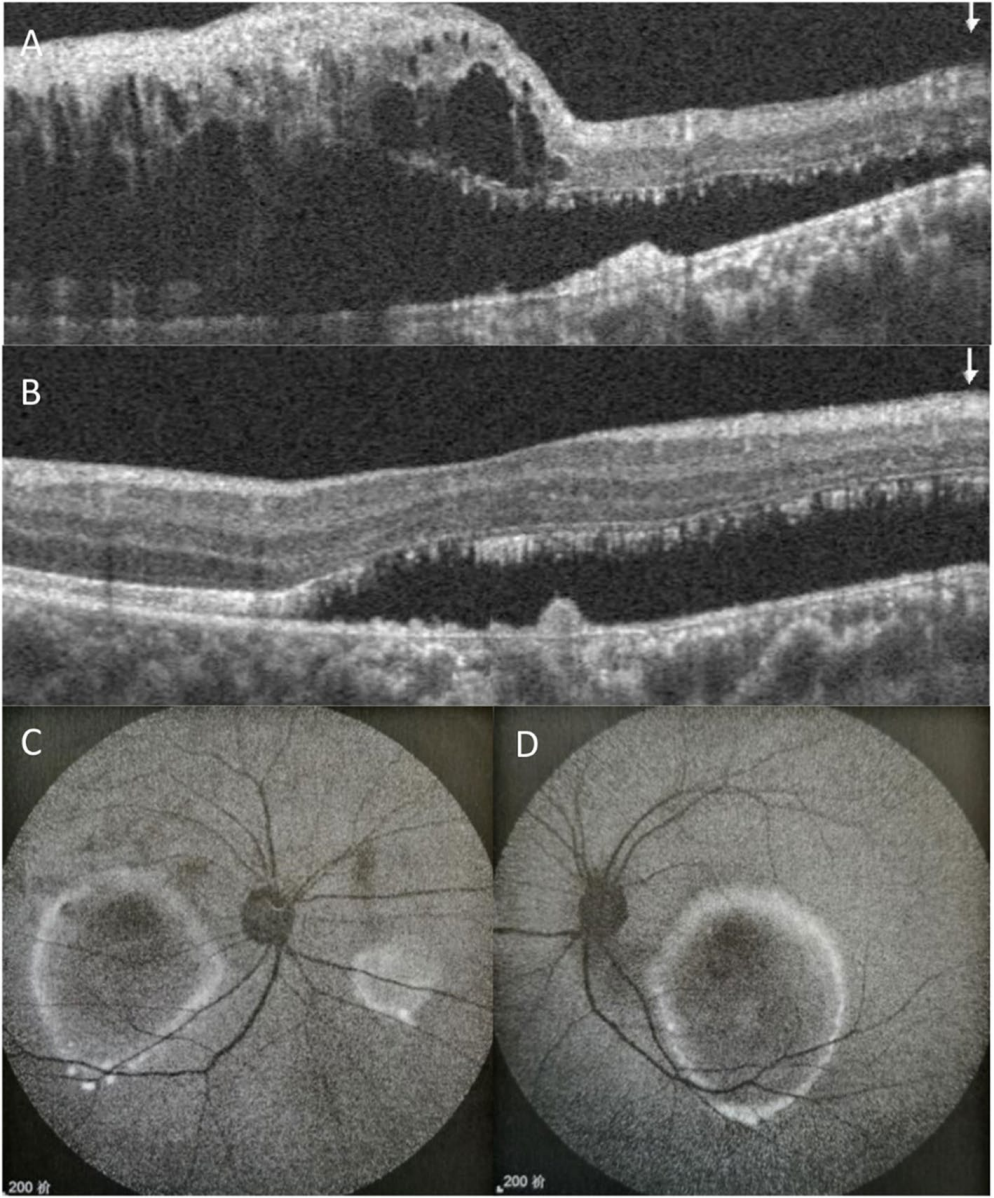
Fundus images of the patient. **A**, **B** Optical coherence tomography imaging at the first visit show subretinal fluid and high-reflective deposition on the retinal pigment epithelial (RPE) layer in both eyes and cystoid macular edema in the right eye. **C**, **D** Autofluorescent images demonstrating multiple autofluorescent deposits with hypofluorescence in the lesion and hyperfluorescence of the surrounding area

It is worth noting that fundus examination revealed pale-yellow deposits in the macula with subretinal fluid in both eyes, besides vascular changes and intraretinal haemorrhages in superior temporal capillaries trajectory in the right eye (Fig. 1E, F). OCT revealed significant accumulation of subretinal fluid, macular cystoid edema and high-reflective deposition on the retinal pigment epithelial (RPE) layer in the right eye. Additionally, fluid under the retinal neurosensory layer (RNFL) with vitelliform lesions (chorioretinal hypertrophic scarring and disruption at the RPE layer) was observed in the left eye (Fig. 2A, B).

**Fig. 3:**
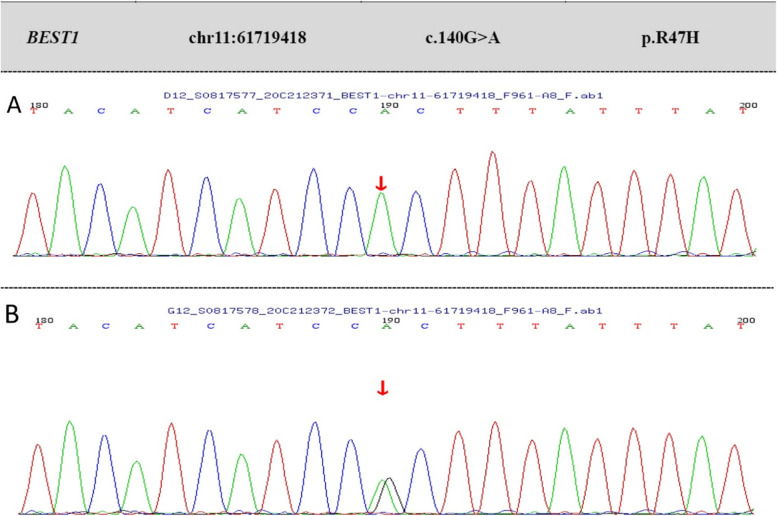
Bidirectional Sanger sequencing of the patient and his son. **A** The top panel shows a homozygotic mutation, c.140G > A (p.R47H), in the patient. **B** The bottom panel shows a heterozygous mutation of c.140G > A in his son

Autofluorescent images demonstrated hypofluorescence in the lesion and hyperfluorescence of the surrounding area in the macula, which is similar to Best vitelliform macular dystrophy (BVMD) (Fig. 2C, D). Subsequently, fluorescent sequence analysis revealed an autosomal recessive inheritance of the chrll:61,719,418 (c.140G > A, p.R47H) homozygous mutation in BEST1 (Fig. 3). However, in most ARBs, fundus autofluorescence does not show typical autofluorescent multifocal deposition [4]. Thus, genetic testing confirmed the diagnosis of ARB.

**Fig. 4:**
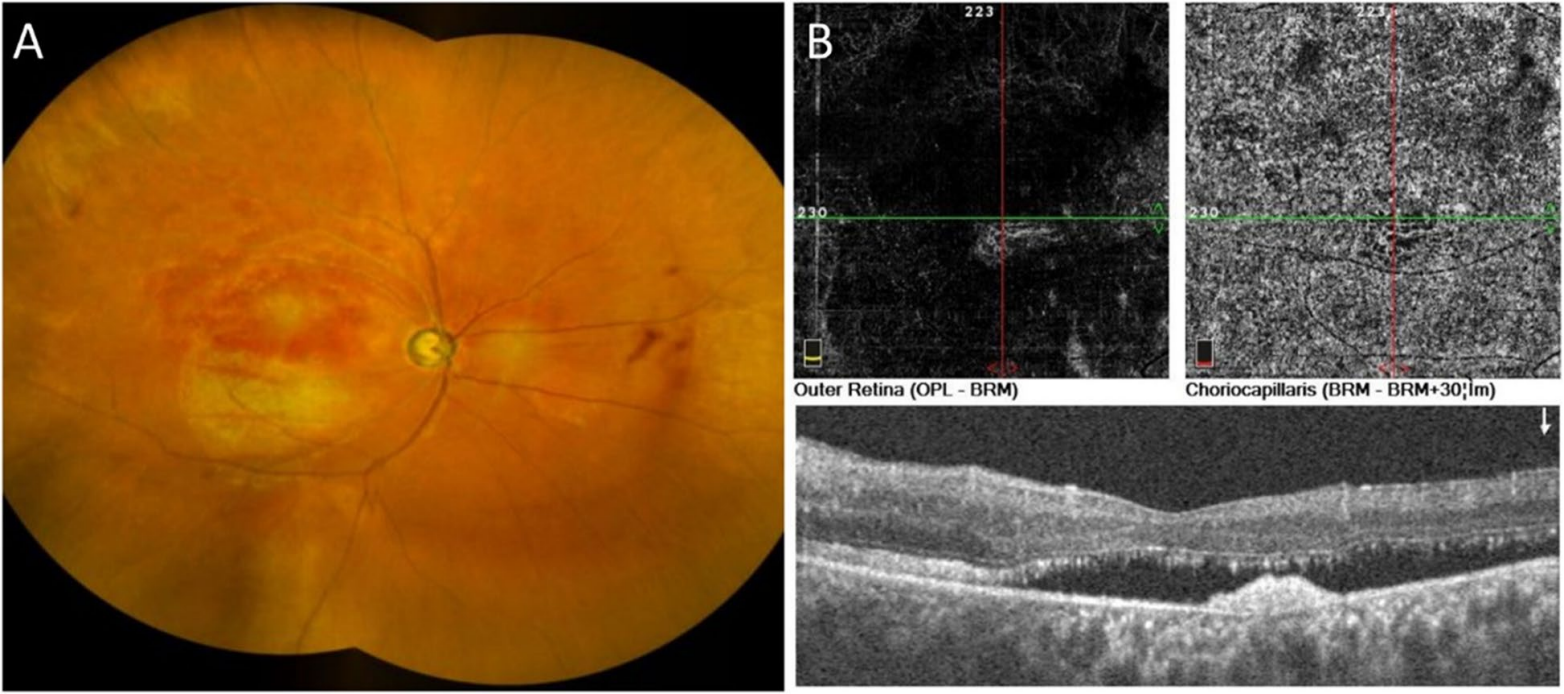
One month after intravitreal ranibizumab. **A** Fundus photograph showing hemorrhage remission. **B** Optical coherence tomography imaging showed that the macular cystoid edema resolved, but the subretinal fluid persisted on the RPE

Finally, the patient was diagnosed with BRVO, ACG, and ARB. Intravitreal ranibizumab 0.5 mg was administered in combination with three drugs to lower IOP in the right eye. At 1-month postinjection, the BCVA improved to 0.3. On OCT, the intraretinal fluid (IRF) gradually resolved and cleared in the right eye. However, the subretinal fluid didn't decreased (Fig. 4). IOP was maintained at 13 mmHg thereafter but required continued use of eyedrops, Travatan® (Travoprost, 0.004%) qn, Mikelan (carteolol hydrochloride, 2%) bid and Azopt (brinzolamide, 10 mg/mL) bid.

## Discussion and conclusion

Homozygous or compound heterozygous mutations of the BEST1 gene caused ARB that are inherited in an autosomal recessive pattern. Homozygous patients had the shortest AL, shallowest ACD, and highest IOP compared with patients with compound heterozygous mutations and heterozygous mutations and those without mutations of BEST1. ARB is strongly associated with anterior segment abnormalities and increased susceptibility to ACG. Approximately 50% of ARB patients also had ACG [5]. Boon et al. declared that ARB may have a dysgenesis of the anterior segment that additionally affects the trabecular meshwork [6]. Therefore, in this case, ACG was likely associated with ARB in this case.

BVMD is characterized by vitelliform lesions that typically occur at the macula. However, ARB is associated with subretinal deposits that occur predominantly outside the macula, mainly at the posterior pole and along the vascular arcades. In Chinese patients, ARBs display multifocal subretinal yellowish deposits/flecks in the macula or retinal vascular arcades along with foveal cystoid macular edema [4]. In this case, the central yellowish subretinal depositions bore some resemblance to the scrambled-egg (the vitelliruptive) stage of BVMD.

Mutation c.140G > A (p.R47H) in Best1 has been reported in ARB, BVMD and AVMD [7, 8]. In this case, the patient had no relative family history, and his parents and his son had no significant clinical symptoms. Although the parents’ DNA was not available, his son had a heterozygous mutation at the same site. Therefore, we assumed it was an autosomal recessive disorder.

The RPE interdigitates with the outer segments of photoreceptors and is important in the maintenance of outer retina homeostasis. ARB is caused by mutations of the BEST1 gene in the RPE. Therefore, we will expect that an anti-VEGF agent would be beneficial for repairing macular cystoid oedema in patients suffering from RVO but not for relieving fluid in the outer layer effectively in these cases, as seen after the first intravitreal dose in our patient and presumably expected in further doses.

It is well known that glaucoma is associated with the risk of RVO. Anatomic changes of the glaucomatous optic nerve might be associated with the pathogenesis of RVO. Meanwhile, individuals with glaucoma, regardless of primary angle closure glaucoma or primary open angle glaucoma, have narrower retinal vessels that healthy individuals [9]. The vascular etiology of glaucoma is likely to facilitate RVO development. However, a stronger association was found between ACG and central retinal vein occlusion, and the frequency of PAC and PACG in BRVO was similar to the value in the general population [10]. Thus, there is a plausible relationship between ACG and BRVO.

A close relationship has been recognized between RVO and glaucoma. RVO, especially CRVO, frequently induces neovascular glaucoma (NVG) [11]. However, this patient had a short course of disease, and he gained an obvious visual field defect and an enlarged optic disc. The limitation of our study was the lack of FFA. Even though FFA was not conducted, no significant neovasculature was found in the anterior segment, including the chamber angle. Therefore, NVG secondary to BRVO could be ruled out. More than likely, ACG may be relevant to ARB.

In conclusion, we assume that the ACG was caused by ARB, while BRVO might be a coincidence in this case.

## Data Availability

Original data are included in this published article. Gene data can be obtained from sources as follows.Sentieon: https://www.sentieon.com/. CNVkit: https://cnvkit.readthedocs.io/en/stable/. ANNOVAR: http://annovar.openbioinformatics.org/en/latest/. Wang K, Li M, Hakonarson H. ANNOVAR: functional annotation of genetic variants from high-throughputsequencing data. Nucleic Acids Res. 2010; 38: e164. 1000 genome: http://www.1000genomes.or/. EVS: http://evs.gs.washington.edu/EVS. dbSNP: http://www.ncbi.nlm.nih.gov/projects/SNP/. EXAC: http://exac.broadinstitute.org/. HGMD: http://www.biobase-international.com/product/hgmd. SIFT: http://sift.jcvi.org/. PolyPhen-2: http://genetics.bwh.harvard.edu/pph2/. MutationTaster: http://www.mutationtaster.org/. GERP +  + : http://mendel.stanford.edu/SidowLab/downloads/gerp/index.html. SPIDEX: http://www.deepgenomics.com/spidex. Sentieon software was used form Oct,2020.

## Abbreviations

ACD: Anterior chamber depth
ACG: Angle-closure glaucoma
ARB: Autosomal recessive bestrophinopathy
BRVO: Branch retina vein occlusion
BVMD: Best vitelliform macular dystrophy
IOP: Intraocular pressure
NVG: Neovascular glaucoma
RNFL: Retinal neurosensory layer
RPE: Retinal pigment epithelial
RVO: Retina vein occlusion
